# Chemical Differentiation and Quantitative Analysis of Different Types of *Panax* Genus Stem-Leaf Based on a UPLC-Q-Exactive Orbitrap/MS Combined with Multivariate Statistical Analysis Approach

**DOI:** 10.1155/2018/9598672

**Published:** 2018-05-03

**Authors:** Lele Li, Yang Wang, Yang Xiu, Shuying Liu

**Affiliations:** Jilin Ginseng Academy, Changchun University of Chinese Medicine, Changchun 130117, China

## Abstract

Two quantitative methods (−ESI full scan and −ESI PRM MS) were developed to analyze ginsenosides in ginseng stem-leaf by using UPLC-Q-Exactive Orbitrap/MS. By means of −ESI PRM MS method, the contents of eighteen ginsenosides in Asian ginseng stem-leaf (ASGSL) and American ginseng stem-leaf (AMGSL) were analyzed. The principal component analysis (PCA) model was built to discriminate Asian ginseng stem-leaf (ASGSL) from American ginseng stem-leaf (AMGSL) based on −ESI PRM MS data, and six ginsenosides (F11, Rf, R2, F1, Rb1, and Rb3) were obtained as the markers. To further explore the differences between cultivated ginseng stem-leaf and forest ginseng stem-leaf, the partial least squares-discriminant analysis (PLS-DA) model was built based on −ESI full scan data. And twenty-six markers were selected to discriminate cultivated ginseng stem-leaf (CGSL) from forest ginseng stem-leaf (FGSL). This study provides reliable and effective methods to quantify and discriminate among different types of ginseng stem-leaf in the commercial market.

## 1. Introduction

Asian ginseng (*Panax ginseng* C. A. Meyer) and American ginseng (*P. quinquefolium* L.) are two different medicinal herbs highly valued all over the world [1–4]. Asian ginseng (ASG) has been used to cure various diseases for thousands of years in Asian countries [5]. Recently, American ginseng (AMG) has also been well known in Asian countries because of its therapeutic functions [2]. These two related and similar-looking functional herbs, however, show certain differences in their biological activities and pharmacological effects. The root of *Panax* genus is the most commonly used part of the plant. And the stem-leaf is attracting more and more attention because of its similar pharmacological activities to the root [6, 7]. The quality and chemical composition of ginseng vary widely depending on the species, variety, geographical origin, cultivation method, environment, and harvesting time [8]. According to the cultivation environment, ASG could be divided into cultivated ginseng (CG) and forest ginseng (FG). The former is cultivated in artificial conditions and grown for 3–7 years, while the latter is produced by sowing seeds in forest and grown in natural environment for over 10 years. In the commercial market, the specifications and grades of commercial products are numerous, and the prices fluctuate widely with the types of ginseng. Since there is no practical criterion for differentiating different CG, FG, and AMG, it is valuable to find analytical markers and provide methods for discriminating different types of ginseng. Much effort has been devoted to the quantitative and qualitative analysis of ginseng roots which are cultivated in different areas and ages [9]. However, little is known about the difference of stem-leaf in chemical compositions between different types of *Panax* genus.

Ginsenosides are the major pharmacologically active constituents of *Panax* genus [10, 11]. They are considered to be responsible for the activities of antioxidant, anti-inflammatory, antiapoptotic, and immunostimulant properties of ginseng [12, 13]. More than 70 ginsenosides have been isolated and identified in *Panax* genus [14]. Few analytical methods, including high-performance liquid chromatography (HPLC) [15], have been developed to exclusively determine ginsenosides in stem-leaf. However, in most of these methods, ginsenosides in minor or trace amounts cannot be detected due to the limited resolution and sensitivity of the detectors. Comparing with HPLC, LC-electrospray-mass spectrometry (ESI-MS) can achieve much higher sensitivity and selectivity and quantify chemical markers in the complex matrixes with only a small amount of sample. Q-Exactive Orbitrap mass spectrometry, with extremely high resolution, sensitivity, and mass accuracy, is a powerful technique for the detection of ginsenosides, especially for those in minor amounts [16, 17]. It provides multiple scan modes, including ESI parallel reaction monitoring (PRM) and ESI full scan MS, which are efficient for the quantification of ginsenosides [18]. Compared with the full scan mode, the PRM mode shows better sensitivity and selectivity without establishing the baseline chromatographic separation of target analytes, but the PRM mode only can be carried out by using standards to achieve accurate quantification. Ginsenosides could be detected in both positive and negative ion modes [19]. Their ionization efficiency and limit of detection are different in each mode. Therefore, it is necessary to evaluate the quantitative performance using PRM in both positive and negative ion modes.

LC-ESI-MS combined with multivariate statistical analysis has been widely applied for the systematic identification and quantification of all metabolites in a given organism or biological sample in the study of metabolomics [9, 20]. The enhanced resolution provided by mass spectrometry, along with powerful chemometric software, allows the simultaneous determination and comparison of thousands of chemical entities [21, 22]. It also has been used to differentiate ginsengs which were cultivated in different areas [23]. In the present study, a UPLC-Q-Exactive Orbitrap/MS method was developed to accurately quantify and identify the ginsenosides extracted from forest ginseng stem-leaf (FGSL), cultivated ginseng stem-leaf (CGSL), and American ginseng stem-leaf (AMGSL).

Both −ESI PRM MS and +ESI PRM MS were validated [24], then the −ESI PRM MS was chosen to quantify the ginsenosides in FGSL, CGSL, and AMGSL. The multivariate statistical analysis was further employed to differentiate the *Panax* genus stem-leaf of various types and also to discover the chemical markers in terms of the detected ginsenosides.

## 2. Materials and Methods

### 2.1. Chemicals and Materials

HPLC-grade methanol (MeOH), acetonitrile (ACN), and formic acid were obtained from TEDIA (Fairfield, OH, USA). Ultrapure water was filtered through a Milli-Q system (Millipore, Billerica, MA, USA). Other reagents were analytical grade. Notoginsenoside R1, notoginsenoside R2, ginsenoside Rb1, ginsenoside Rb2, ginsenoside Rb3, ginsenoside Rc, ginsenoside Rd, ginsenoside Re, ginsenoside Rf, ginsenoside Rg1, ginsenoside Rg2, ginsenoside Rg3, ginsenoside Rh1, ginsenoside Rh2, ginsenoside Ro, ginsenoside F1, ginsenoside F2, and pseudoginsenoside F11 standards (they were represented by R1, R2, Rb1, Rb2, Rb3, Rc, Rd, Re, Rf, Rg1, Rg2, Rg3, Rh1, Rh2, Ro, F1, F2, and F11, resp. in the rest sections) were purchased from Nanjing Zelang Co. (purity ≥ 98%, Nanjing, China). Their chemical information is shown in Table 1. AMGSL (10 samples), CGSL (11 samples), and FGSL (11 samples) were collected from Jilin and Heilongjiang provinces. And detailed information of them is shown in Table 2.

### 2.2. Sample Preparation

A certain amount of Noto R1, Noto R2, Rb1, Rb2, Rb3, Rc, Rd, Re, Rf, Rg1, Rg2, Rg3, Rh1, Rh2, Ro, F1, F2, and F11 were dissolved in methanol, respectively, to get eighteen standard solutions. They were mixed and diluted by 70% methanol to get stock solutions and concentrations of each standard in stock solutions are shown in Table 1. Then the stock solutions were diluted to be 1.0, 3.3, 10.0, 33.3, 100.0, 333.3, 1000.0, 3333.3, 10,000.0, 33,333.3, 100,000.0, and 333,333.3 fold dilutions by 70% methanol, respectively, for method validation. All the prepared solutions were stored at 4°C.

The stem-leaf of *Panax* genus samples were dried and pulverized to powder and then passed through a 10-mesh sieve. The obtained powder was weighed (0.1 g) and extracted with 5 mL of 70% methanol in an ultrasonic waterbath for 60 min. The extract was filtered through a syringe filter (0.22 *μ*m) and injected directly into the UPLC system.

### 2.3. Method Validation

An external calibration method was used for the quantitative analysis. And one of the CGSL samples was used in method validation. The linear calibration curves were constructed by plotting the concentrations of six to eleven mixed standard solutions against the corresponding peak areas. The limit of detection (LOD) and limit of quantification (LOQ) were measured with the signal-to-noise ratio of 3 and 10, respectively. The precision was performed by analysis of the standard solution six times and the results were expressed as the relative standard deviation (RSD). For repeatability test, six independent sample solutions were prepared using the procedures described in the last section. The recovery of this method was achieved using the standard addition method. Different concentration levels of the standard solutions were added to the sample six times. The average recoveries were determined by the following formula:

(1) $$\begin{array}{c} \begin{array}{l} \text{Recovery}\left(\%\right)=\frac{\left(\text{observed amount}-\text{original amount}\right)}{\text{spiked amount}}\times 100\%,\\ \text{RSD}\left(\%\right)=\left(\frac{\text{SD}}{\text{mean}}\right)\times 100\%. \end{array} \end{array}$$

### 2.4. UPLC-Q-Exactive Orbitrap/MS Analysis

Chromatographic separation was performed on an Ultimate 3000 system (Dionex, Sunnyvale, CA, USA) coupled with Golden C18 column (2.1 × 50 mm, 1.9 *μ*m; Thermo Fisher, USA). The column temperature was maintained at 30°C, and the mobile phases A and B were water with 0.1% formic acid and acetonitrile, respectively. The gradient elution was programmed to get the ginsenoside profile, and the proportion of acetonitrile (B) was increased from 19% to 19% (0–5 min), 19–28% (5–12 min), 28–40% (12–22 min), 40–85% (22–24 min), and finally adjusted from 85% to 19% (24–25 min) and maintained at 19% for five minutes. The injection volume was 5 *μ*L and the flow rate was 0.3 mL/min.

Mass spectrometric detection was carried out on a Q-Exactive Orbitrap/MS (Thermo, San Jose, CA, USA) equipped with an electrospray ionization (ESI) ion source operated in the positive or negative ion mode. The parameters of ion source were set as follows: sheath gas flow of 40 Arb, aux gas flow of 12 Arb, and sweep gas flow of 1 Arb. The S-Lens RF level was 55%. Capillary voltage was set to +3.5 kV or −3.5 kV with a capillary temperature of 333°C and an aux gas heater temperature of 317°C. Full scan MS data were acquired at the centroid mode from *m*/*z* 200 to 2000, 70,000 resolution, automatic gain control (AGC) target of 1*e*6, and maximum injection time (IT) of 100 ms. The mass range of MS^2^ spectra varies depending on the precursor ions. The precursor and product ions used for the quantification in the PRM mode are listed in Table 3, together with the corresponding normalized collision energy (NCE). PRM MS data were acquired at the centroid mode, 17,500 resolution, AGC target of 1*e*5, and maximum IT of 50 ms.

### 2.5. Multivariate Statistical Analysis

For the −ESI PRM MS, the contents of eighteen ginsenosides in ginseng stem-leaf were used as a dataset containing sample code, ginsenoside, and content. For the −ESI full scan MS, the raw data of samples were processed by the Sieve software (version 2.1, Thermo, San Jose, CA, USA), which could detect the mass, retention time, and intensity of the peaks in each chromatogram. The maximum retention time shift was set at 0.25 min and the *m*/*z* width was 10 ppm to align the features. The base peak minimum intensity and background were set at 10^5^ and 3, respectively. After being aligned, the intensity of each ion was normalized by the total ion intensity of each chromatogram. The resultant dataset, containing *m*/*z* value @ retention time, the normalized intensity and the sample code, was used to perform the multivariate statistical analysis. Then, both the datasets were saved as .csv files and imported into SIMCA-P software 11.5 (Umetrics, Umea, Sweden) to conduct the multivariate statistical analysis including principal component analysis (PCA) and partial least squares-discriminant analysis (PLS-DA). In the PLS-DA model, ions with variable importance in projection (VIP) 1 and VIP 2 values larger than 1 were highlighted and were further filtered by Student's *t*-test (SPSS19.0, Chicago, IL, USA). The components with *p* < 0.05 were considered significant and were selected as analytical markers.

## 3. Results and Discussion

### 3.1. Optimization of LC-MS Conditions

**Figure 1:**
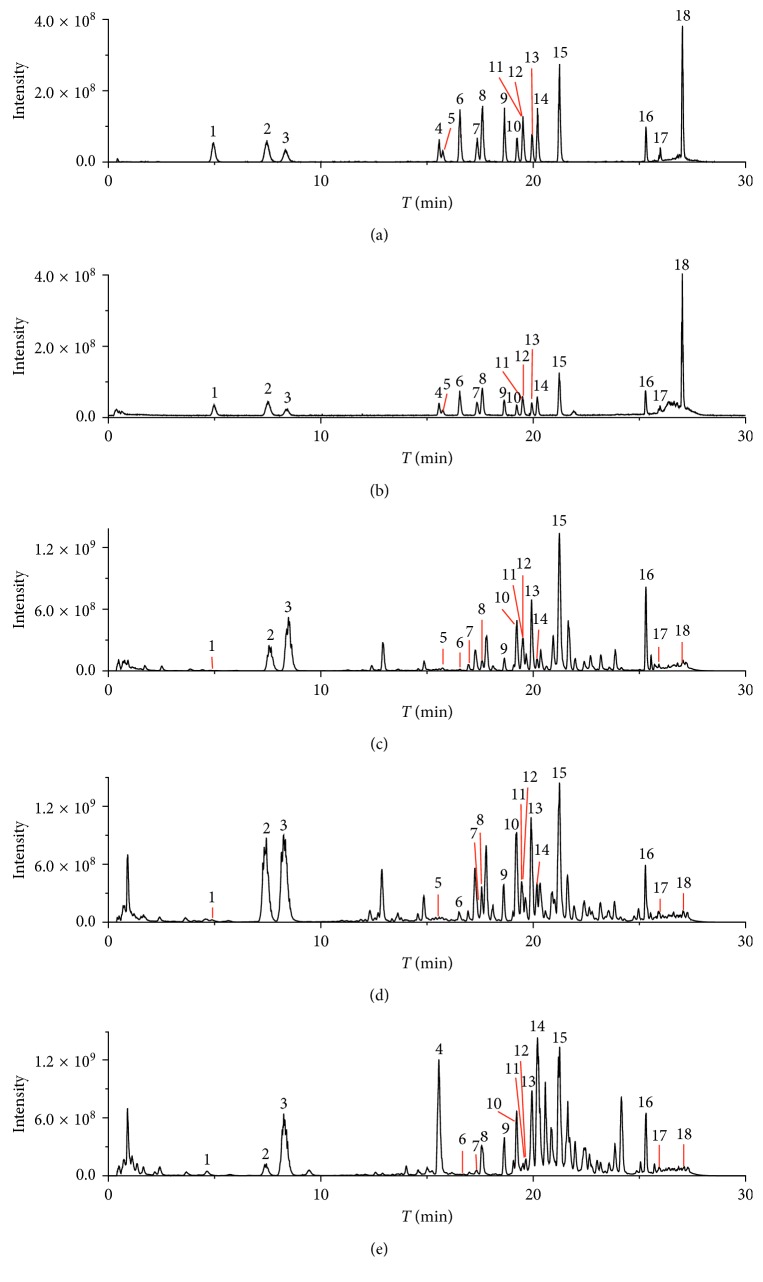
Total ion current chromatograms of eighteen ginsenoside standards based on (a) −ESI full scan and (b) +ESI full scan MS; Total ion current chromatograms of (c) FGLS, (d) CGLS and (e) AMGLS based on −ESI full scan MS (1 notoginsenoside R1; 2 ginsenoside Rg1; 3 ginsenoside Re; 4 pseudoginsenoside F11; 5 ginsenoside Rf; 6 notoginsenoside R2; 7 ginsenoside Rh1; 8 ginsenoside Rg2; 9 ginsenoside Rb1; 10 ginsenoside Rc; 11 ginsenoside F1; 12 ginsenoside Ro; 13 ginsenoside Rb2; 14 ginsenoside Rb3; 15 ginsenoside Rd; 16 ginsenoside F2; 17 ginsenoside Rg3; 18 ginsenoside Rh2).

As shown in Figures 1(a) and 1(b), the total ion chromatograms (TIC) of eighteen ginsenoside standards were obtained in −ESI full scan and +ESI full scan MS. Most of the standards were separated distinctly within 30 min, with an exception of Rf and F11. Rf and F11 were structural isomers and the characteristic components in Asian ginseng and American ginseng, respectively. Theoretically, they cannot be present in one ginseng sample. Although they exhibited a poor chromatographic separation in the full scan mode, their quantification could be achieved in PRM mode, in which they were separated by their distinct ion pairs.

**Figure 2:**
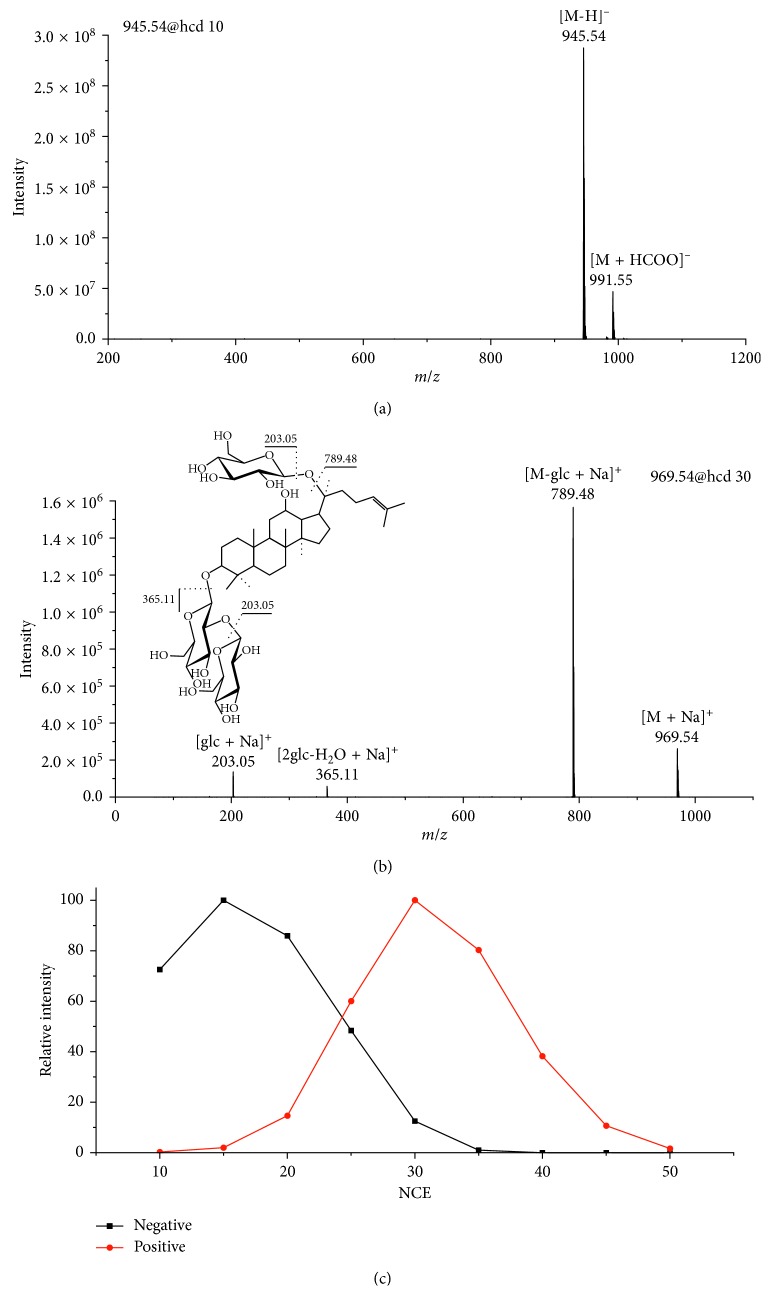
MS^2^ spectra of (a) [M + HCOO]^−^ and (b) [M + Na]^+^ for Rd. (c) The signal intensity of product ions for Rd with respect to the NCE (glc represents glucose).

The ion pairs used for the quantitative analysis of ginsenosides were firstly optimized. Generally, the base peak in the full scan spectra (usually the adduct ion) was taken as the precursor ion, while the base peak in the MS^2^ spectra was selected as the product ion. Taking Rd as an example, the [M + HCOO]^−^ ion at *m*/*z* 991.55 was the main adduct ion of Rd in the −ESI full scan MS and was chosen as the precursor ion. Its corresponding MS^2^ spectrum is shown in Figure 2(a). The [M-H]^−^ ion at *m*/*z* 945.54 was the base peak in the MS^2^ spectrum and was selected as the quantitative product ion. Similarly, the [M+Na]^+^ at *m*/*z* 969.54 in the +ESI full scan MS and [M-glc + Na]^+^ at *m*/*z* 789.48 ions in the MS^2^ spectrum were chosen as the precursor and product ions, respectively. The NCE which ranged from 10% to 50% was further optimized to get the maximum intensity of the product ions. As shown in Figure 2(c), the intensity of the negative ion at *m*/*z* 945.54 and positive ion at *m*/*z* 789.48 was maximized at NCE of 15% and 30%, respectively.

**Figure 3:**
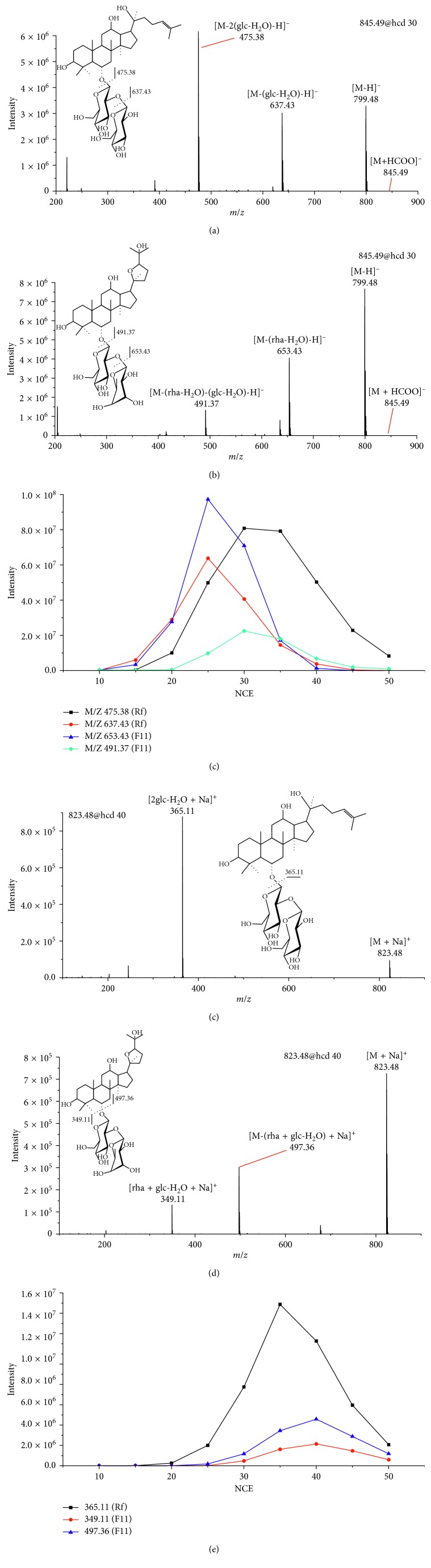
MS^2^ spectra of [M + HCOO]^−^ for (a) Rf and (b) F11 in the negative mode; MS^2^ spectra of [M + Na]^+^ for (d) Rf and (e) F11 in the positive mode. The signal intensity of product ions for Rf and F11 with respect to the NCE in the negative (c) and positive (f) modes (glc represents glucose and ara represents arabinose).

To perform the quantification of isomeric ginsenosides Rf and F11, their PRM conditions were optimized. The precursor ions of Rf and F11 were the [M + HCOO]^−^ ion at *m*/*z* 845.49 in the −ESI full scan MS and the [M + Na]^+^ ion at *m*/*z* 823.48 in the +ESI full scan MS. As shown in Figures 3(a) and 3(b), Rf and F11 had the same [M-H]^−^ ion at *m*/*z* 799.48 and distinct product ions at *m*/*z* (475.38, 637.43) and (491.37, 653.43), respectively, which were obtained by the successive losses of the two saccharide moieties. These distinct ions could be used as the characteristic product ions for their differentiation and quantification. Therefore, the ion at *m*/*z* 475.38 and 653.43 were selected as the product ions of Rf and F11, respectively, as their intensities were higher than the counterparts. In addition, the NCE was optimized to be 30% and 25% for Rf and F11 (Figure 3(c)). In the positive ion mode, the fragment ions were also unambiguously identified, as shown in Figures 3(d) and 3(e). All the optimized PRM conditions are shown in Table 3.

### 3.2. Validation of the Method

As shown in Table 4, the precisions for all the eighteen ginsenosides from two scan modes were in the range from 1.17% to 4.67%, while the repeatability ranged from 2.18% to 10.05%. The results showed that the developed UPLC-Q-Exactive Orbitrap/MS method had a good precision and repeatability for quantifying ginsenosides in all the tested modes.

The LOD and LOQ of eighteen ginsenosides are shown in Table 5. In total, the lower LOD and LOQ were obtained in −ESI PRM MS instead of +ESI PRM MS. In addition, the −ESI PRM MS has the lower LOD and LOQ less than 0.0276 and 0.092 *μ*g/mL for all the ginsenosides.

As shown in Table 6, all the compounds showed good linearity (*r* ≥ 0.99) when the method was operated in the negative ion mode. The concentration range could reach up to 3 orders of magnitude, indicating the developed method a good capability to quantify the ginsenosides. Only a few ginsenosides showed good linearity in +ESI PRM MS. It is observed that the linearity of ginsenosides detected in the negative ion mode was better than that in the positive ion mode.

The recoveries detected in both the modes were in the range from 65.42% to 137.71% with RSD less than 10.60%, as shown in Table 7.

The validation results indicated that the −ESI PRM MS was better than +ESI PRM MS in LOD, LOQ, linearity, concentration range, and mean recovery. Therefore, the described −ESI PRM MS method was subsequently applied to the analysis of all the samples. The developed UPLC-Q-Exactive Orbitrap/MS methods showed lower LOD and LOQ, larger concentration range than HPLC methods [18, 25].

### 3.3. Quantification Analysis Based on −ESI PRM MS Data

The TIC plots of the extracted AMGSL, CGSL, and FGSL are shown in Figures 1(c)–1(e). The differences between AMGSL and Asian Ginseng stem-leaf (ASGSL) could be clearly observed, as Rf and F11 were only present in ASGSL and AMGSL, respectively. The contents of eighteen ginsenosides in every type of samples were detected using the developed PRM/− method, and the quantitative results are shown in Table 8. Excepting Rf and F11, AMGSL and ASGSL also had large differences in the contents of R2, Rb3, and F1. AMGSL contained less R2, F1 but more Rb3 than ASGSL. For CGSL and FGSL, it was found that the contents of eighteen ginsenosides were very similar. Therefore, it is difficult to discriminate CGSL from FGSL just using the data of contents of eighteen ginsenosides. To discriminate among different ginseng stem-leaves and find analytical markers, the multivariate statistical analysis was performed in the next section.

### 3.4. Multivariate Statistical Analysis

**Figure 4:**
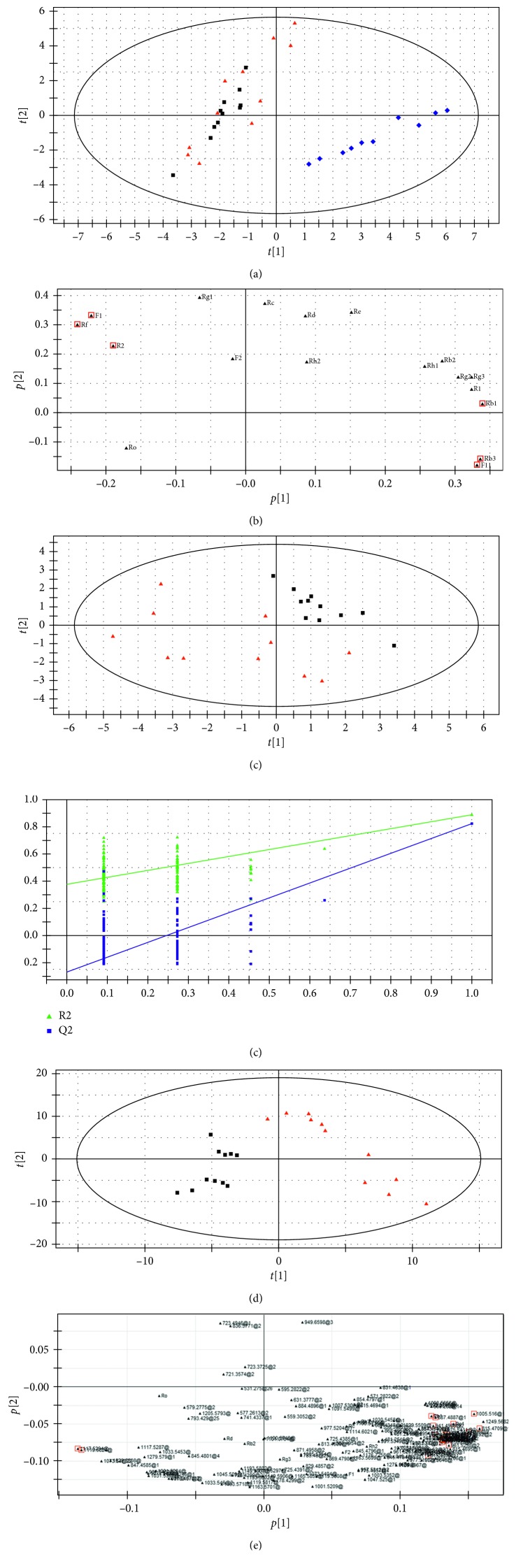
(a) Score plot and (b) loading plot of PCA model based on data of eighteen ginsenoside contents in CGLS, FGLS, and AMGLS. (c) Score plot of PLS-DA model based on data of eighteen ginsenoside contents in CGLS and FGLS. (d) Permutation test, (e) score plot, and (f) loading plot of PLS-DA model based on −ESI full scan MS data (▲, CGLS; ■, FGLS; ◆, AMGLS).

The dataset of the ginsenoside contents was subjected to the multivariate statistical analysis to differentiate the samples from different origins and to discover the chemical markers. Firstly, a PCA mode, an unsupervised pattern recognition technique, was established with *R*^2^(*X*) (cum) = 0.850 and *Q*^2^ (cum) = 0.561. As shown in Figure 4(a), the samples are separated distinctly into two groups. CGSL and FGSL were clustered together, suggesting their similar content of ginsenosides. And the clear separation between AMGSL and ASGSL was achieved, indicating the significant difference between these two species. To reveal the ginsenosides that contribute most to the separations of samples from different origins, the loading plot was used to select the chemical markers. As shown in Figure 4(b), the ginsenosides far from the origin were considered to contribute most to the separation and were selected as chemical marker candidates. These ginsenosides were further filtered by Student's *t*-test. The variables with contents which are statistically significant different (*p* < 0.05) between two groups were selected as the analytical markers. Finally, six ginsenosides were obtained as the markers and marked with red boxes in the loading plot. They were F11, Rf, R2, F1, Rb1, and Rb3 respectively.

To further explore the differences of targeted ginsenosides present in ASGSL samples, the PLS-DA model [26] was established by setting CGSL as group I and FGSL as group II. The PLS-DA scores plot is established with the model parameters of *R*^2^(*Y*) = 0.742 and *Q*^2^ (cum) = 0.615 and shown in Figure 4(c). A clear separation is still not observed, since the limited ginsenosides could not provide enough characteristic information for their differentiation.

Therefore, the PLS-DA model was established based on the data obtained in −ESI full scan MS method was used to separate CGSL and FGSL. As shown in Figure 4(e), clear separations are observed with *R*^2^(*Y*) = 0.890 and *Q*^2^ (cum) = 0.824, suggesting good fitness and prediction ability of the established PLS-DA model. Permutation test (*n*=200) was further performed to validate the model. No overfitting was found because the permutated R2 and Q2 values on the left are lower than the original point on the right (Figure 4(d)). These results indicated that the established PLS-DA mode has the high goodness of fit and predictability. Then loading plot (Figure 4(f)) and VIP were used to reveal the potential analytical markers that contribute most to the separation between CGSL and FGSL. After *t*-test, twenty-six chemical components showed statistical differences between groups and considered to be the chemical markers for the separation of ASGSL and marked with red boxes in the loading plot (Figure 4(f)).

**Figure 5:**
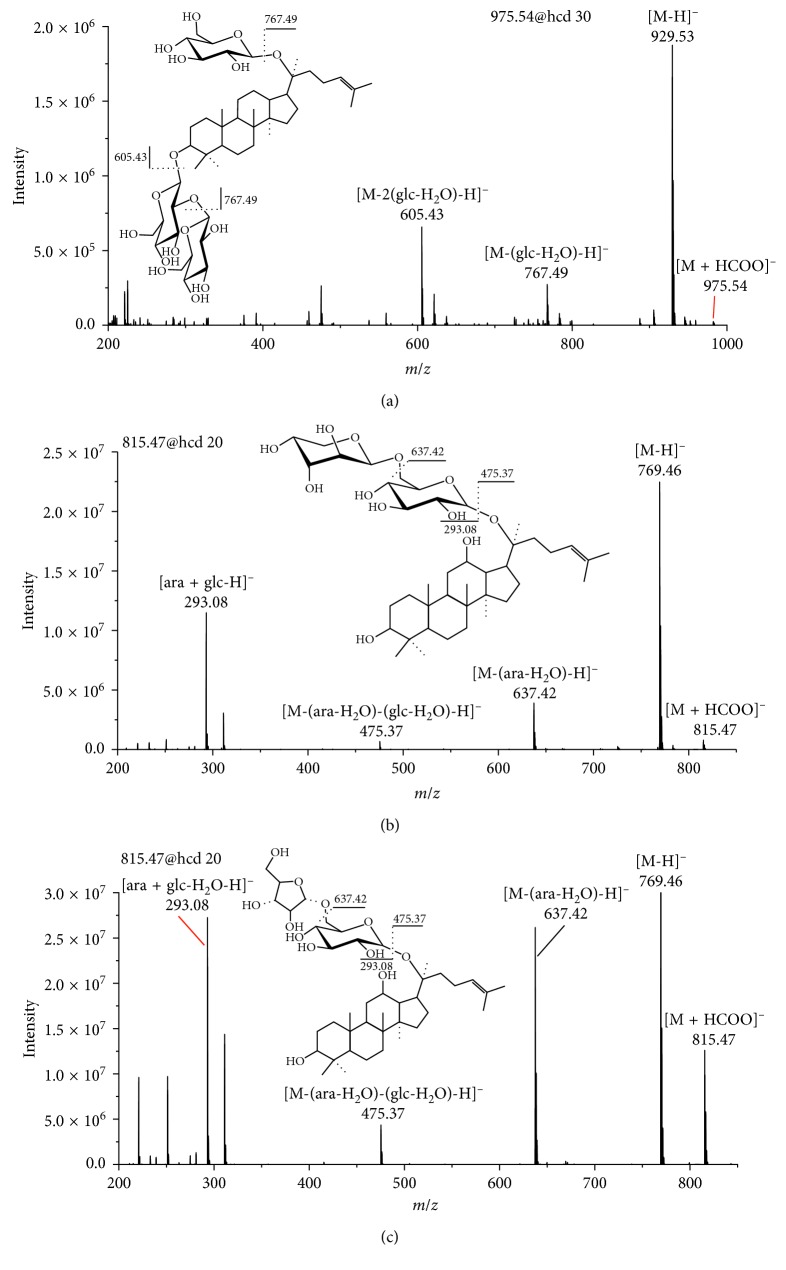
MS^2^ spectra of [M + HCOO]^−^ for (a) Vina-ginsenoside R3, (b) ginsenoside F3, and (c) ginsenoside F5 in the negative mode. (The glc represent glucose and the ara represent arabinose.)

Excepting Rb1, Rg1, and R1, the identification of rest analytical markers was conducted by means of accurate mass-to-charge ratio and MS^2^ information obtained by MS. The marker 975.54@20.84 was assigned to Vina-ginsenoside R3 [27] whose MS/MS spectrum is shown in Figure 5(a). The [M + HCOO]^−^ ion of Vina-ginsenoside R3 at *m*/*z* 975.54 showed the characteristic product ions at *m*/*z* 767.49 and 605.43. The markers 815.47@18.11 and 815.47@17.26 were assigned to ginsenoside F3 and ginsenoside F5, respectively. The fragmentation schemes for the product ions of ginsenoside F3 and ginsenoside F5 are shown in Figures 5(b) and 5(c). The chemical information of analytical markers is shown in Table 9. All the contents of Rg1, Rb1, R1, Vina-ginsenoside R3, ginsenoside F3, and ginsenoside F5 in the FGSL were less than in the CGSL. The reason may be related to cultivation.

## 4. Conclusions

By using UPLC-Q-Exactive Orbitrap/MS, a rapid, simple, and reliable method to simultaneously determine eighteen ginsenosides (R1, R2, Rb1, Rb2, Rb3, Rc, Rd, Re, Rf, Rg1, Rg2, Rg3, Rh1, Rh2, Ro, F1, F2, and F11) in *Panax* genus stem-leaf was first developed and validated. This method provides an excellent quantitative tool for analysis of ginsenosides in stem-leaf due to its high capacity, high sensitivity, and high selectivity. F11, Rf, R2, F1, Rb1, and Rb3 are analytical markers which could be used to identified ASGSL and AMGLS. Rf was found only in ASGSL, and F11 was found exclusively in AMGLS, which were similar to the difference between Asian ginseng root and American ginseng root [28]. Furthermore, our results suggest that the approach in the present study could be effectively applied to discriminate CGSL from FGSL. Based on UPLC-Q-Exactive Orbitrap/MS combined with multivariate statistical analysis, a reliable and effective approach aimed to discriminate among different types of *Panax* genus stem-leaf has been successfully developed.

## Figures and Tables

**Table 1:** Chemical information of the eighteen ginsenoside standards.

Compounds	Formula	Accurate mass	Retention time (min)	Concentration (*μ*g/mL)
Noto R1	C_47_H_80_O_18_	932.5345	4.89	288
Noto R2	C_41_H_70_O_13_	770.4816	16.54	222
Rb1	C_54_H_92_O_23_	1108.6029	18.61	108
Rb2	C_53_H_90_O_22_	1078.5924	19.92	162
Rb3	C_53_H_90_O_22_	1078.5924	20.20	240
Rc	C_53_H_90_O_22_	1078.5924	19.21	72
Rd	C_48_H_82_O_18_	946.5501	21.20	372
Re	C_48_H_82_O_18_	946.5501	8.29	222
Rf	C_42_H_72_O_14_	800.4922	15.70	68
Rg1	C_42_H_72_O_14_	800.4922	7.41	252
Rg2	C_42_H_72_O_13_	784.4973	17.60	264
Rg3	C_42_H_72_O_13_	784.4973	25.98	4
Rh1	C_36_H_62_O_9_	638.4394	17.37	114
Rh2	C_36_H_62_O_8_	622.4445	27.02	276
Ro	C_48_H_76_O_19_	956.4981	19.54	180
F1	C_36_H_62_O_9_	638.4394	19.50	150
F2	C_42_H_72_O_13_	784.4973	25.30	120
F11	C_42_H_72_O_14_	800.4922	15.57	72

**Table 2:** The information about growth years and collecting location for all *Panax* genus stem-leaf samples.

Number	Growth years	Collecting location	Number	Growth years	Collecting location
AMGSL-1	3	Suihua city, Heilongjiang province	CGSL-7	4	Suihua city, Heilongjiang province
AMGSL-2	4	Suihua city, Heilongjiang province	CGSL-8	4	Hunchun city, Jilin province
AMGSL-3	3	Jiaohe city, Jilin province	CGSL-9	5	Hunchun city, Jilin province
AMGSL-4	5	Jiaohe city, Jilin province	CGSL-10	5	Hunchun city, Jilin province
AMGSL-5	4	Antu county, Jilin province	CGSL-11	5	Wangqing county, Jilin province
AMGSL-6	3	Jiaohe city, Jilin province	FGSL-1	16	Huadian city, Jilin province
AMGSL-7	4	Jiaohe city, Jilin province	FGSL-2	14	Huadian city, Jilin province
AMGSL-8	3	Suihua city, Heilongjiang province	FGSL-3	11	Panshi city, Jilin province
AMGSL-9	4	Antu county, Jilin province	FGSL-4	12	Huadian city, Jilin province
AMGSL-10	5	Panshi city, Jilin province	FGSL-5	15	Huadian city, Jilin province
CGSL-1	3	Suihua city, Heilongjiang province	FGSL-6	16	Suihua city, Heilongjiang province
CGSL-2	4	Suihua city, Heilongjiang province	FGSL-7	14	Huadian city, Jilin province
CGSL-3	3	Suihua city, Heilongjiang province	FGSL-8	13	Panshi citiy, Jilin province
CGSL-4	3	Suihua city, Heilongjiang province	FGSL-9	10	Panshi city, Jilin province
CGSL-5	3	Tieli county, Heilongjiang province	FGSL-10	10	Panshi city, Jilin province
CGSL-6	4	Hunchun city, Jilin province	FGSL-11	11	Dongning county, Heilongjiang province

**Table 3:** Results of optimization for product ions and normalized collision energy.

Compounds	Negative			Positive		
	Precursor ion	Product ion	NCE (%)	Precursor ion	Product ion	NCE (%)
Noto R1	977.53	931.53	15	955.52	775.46	30
Noto R2	815.48	769.48	15	793.45	335.10	35
Rb1	1107.60	945.54	25	1131.59	365.11	30
Rb2	1123.59	1077.58	10	1101.58	335.10	35
Rb3	1123.59	1077.58	10	1101.58	335.10	35
Rc	1123.59	1077.58	10	1101.58	335.10	35
Rd	991.55	945.54	15	969.54	789.48	30
Re	991.55	945.54	15	969.54	789.48	30
Rf	845.49	475.38	30	823.48	365.11	40
Rg1	845.49	799.48	15	823.48	643.42	25
Rg2	829.50	783.49	15	807.49	349.11	35
Rg3	829.50	783.49	20	807.49	365.11	35
Rh1	683.44	637.43	15	661.43	203.05	35
Rh2	667.44	621.44	15	645.43	203.05	35
Ro	955.49	793.44	30	979.49	641.40	25
F1	683.44	637.43	15	661.43	203.05	30
F2	829.50	783.49	20	807.49	627.42	30
F11	845.49	653.43	30	823.48	497.36	40

**Table 4:** Precision and repeatability for the eighteen ginsenosides in ginseng stem-leaf.

Compounds	RSD (%) precision (*n*=6)		RSD (%) repeatability (*n*=6)	
	−ESI PRM	+ESI PRM	−ESI PRM	+ESI PRM
R1	2.30	2.34	5.63	2.94
R2	4.31	2.93	7.28	2.73
Rb1	2.44	3.93	4.14	3.31
Rb2	2.89	4.47	4.54	2.90
Rb3	4.50	4.67	3.52	4.05
Rc	3.60	2.42	4.05	2.18
Rd	3.21	1.83	7.14	7.28
Re	2.47	2.04	7.85	5.88
Rf	2.76	2.24	8.56	2.46
Rg1	3.26	2.15	4.98	6.41
Rg2	3.40	1.38	9.30	3.04
Rg3	3.76	2.40	2.89	10.05
Rh1	1.93	2.14	7.32	8.33
Rh2	3.07	2.50	4.11	7.62
Ro	1.18	1.17	4.85	5.04
F1	3.22	2.05	2.43	4.40
F2	2.93	4.07	8.26	7.35
F11	3.02	3.33	4.36	6.24

**Table 5:** LOD and LOQ for the eighteen ginsenosides in ginseng stem-leaf.

Compounds	LOD (*μ*g/mL)		LOQ (*μ*g/mL)	
	−ESI PRM	+ESI PRM	−ESI PRM	+ESI PRM
R1	0.00288	0.0288	0.0096	0.096
R2	0.0222	0.0222	0.074	0.074
Rb1	0.0036	0.0108	0.0108	0.036
Rb2	0.00054	0.0162	0.00162	0.054
Rb3	0.0024	0.024	0.008	0.08
Rc	0.00024	0.0072	0.00072	0.024
Rd	<0.00124	0.0372	0.00124	0.124
Re	0.00074	0.0222	0.00222	0.074
Rf	0.000228	0.0228	0.000684	0.0684
Rg1	0.00252	0.0252	0.0084	0.084
Rg2	0.00088	0.088	0.00264	0.264
Rg3	0.000134	0.00402	0.000402	0.0134
Rh1	0.0038	0.114	0.0114	0.38
Rh2	0.0276	0.0092	0.092	0.0276
Ro	0.0006	0.006	0.0018	0.018
F1	0.0015	0.015	0.015	0.05
F2	0.012	0.004	0.04	0.012
F11	0.00072	0.024	0.0024	0.072

**Table 6:** Calibration curve for the eighteen ginsenosides in ginseng stem-leaf.

Compounds	−ESI PRM			+ESI PRM		
	Calibration curve	Linear range (*μ*g/mL)	*r*	Calibration curve	Linear range (*μ*g/mL)	*r*
R1	*Y* = 3.27018 × 10^7^*X* + 1.27657 × 10^7^	0.0096–288	0.9994	*Y* = 7.12403 × 10^5^*X* + 1.14396 × 10^6^	0.096–28.8	0.9892
R2	*Y* = 2.87993 × 10^7^*X* + 5.33922 × 10^7^	0.074–74	0.9942	*Y* = 1.82435 × 10^5^*X* + 2.53254 × 10^5^	0.074–22.2	0.9897
Rb1	*Y* = 4.44403 × 10^6^*X* + 1.70415 × 10^6^	0.0108–36	0.9985	*Y* = 9.55059 × 10^5^*X* + 1.91469 × 10^6^	0.036–36	0.9850
Rb2	*Y* = 2.47272 × 10^7^*X* + 1.29975 × 10^7^	0.00162–54	0.9978	*Y* = 5.82544 × 10^5^*X* + 1.65322 × 10^6^	0.054–54	0.9846
Rb3	*Y* = 2.15503 × 10^7^*X* + 3.80369 × 10^7^	0.008–80	0.9914	*Y* = 2.89207 × 10^5^*X* + 1.49713 × 10^6^	0.08–80	0.9818
Rc	*Y* = 4.72793 × 10^7^*X* + 1.12854 × 10^7^	0.00072–24	0.9979	*Y* = 8.45948 × 10^5^*X* + 1.38423 × 10^6^	0.024–24	0.9804
Rd	*Y* = 3.37678 × 10^7^*X* + 1.08663 × 10^8^	0.00124–124	0.9815	Y = 3.53253 × 10^5^*X* + 3.07447 × 10^6^	0.124–124	0.9807
Re	*Y* = 3.19849 × 10^7^*X* + 2.13175 × 10^7^	0.00222–222	0.9970	*Y* = 4.69691 × 10^5^*X* + 2.40931 × 10^6^	0.074–74	0.9796
Rf	*Y* = 8.08178 × 10^7^*X* + 9.99727 × 10^6^	0.000684–22.8	0.9993	*Y* = 4.29504 × 10^5^*X* + 5.30340 × 10^5^	0.0684–22.8	0.9906
Rg1	*Y* = 8.38404 × 10^6^*X* + 3.00715 × 10^7^	0.0084–252	0.9958	*Y* = 1.15403 × 10^6^*X* + 2.47 × 10^6^	0.084–25.2	0.9771
Rg2	*Y* = 4.17409 × 10^7^*X* + 1.14333 × 10^7^	0.00264–26.4	0.9974	*Y* = 5.8844 × 10^4^*X* + 4.43358 × 10^5^	0.264–88	0.9879
Rg3	*Y* = 3.47553 × 10^8^*X* + 6.73022 × 10^6^	0.000402–1.34	0.9964	*Y* = 1.58307 × 10^6^*X* + 1.31307 × 10^5^	0.0134–1.34	0.9890
Rh1	*Y* = 3.4999 × 10^6^*X* + 2.45245 × 10^6^	0.0114–38	0.9950	*Y* = 4.7729 × 10^4^*X* + 4.6489 × 10^4^	0.38–11.4	0.9878
Rh2	*Y* = 1.3957 × 10^6^*X* + 1.25926 × 10^6^	0.092–27.6	0.9937	*Y* = 1.45527 × 10^6^*X* + 1.52536 × 10^6^	0.092–27.6	0.9937
Ro	*Y* = 5.2621 × 10^6^*X* + 5.94325 × 10^6^	0.0018–60	0.9925	*Y* = 1.82466 × 10^6^*X* + 2.10478 × 10^6^	0.018–60	0.9819
F1	*Y* = 4.03707 × 10^6^*X* + 1.93111 × 10^6^	0.015–50	0.9981	*Y* = 1.06804 × 10^6^*X* + 2.54794 × 10^5^	0.05–15	0.9982
F2	*Y* = 3.14728 × 10^6^X + 152,642	0.04–12	0.9998	*Y* = 1.48171 × 10^6^*X* + 2.40890 × 10^5^	0.04–12	0.9878
F11	*Y* = 8.90659 × 10^5^*X* + 20,891	0.0024–24	1.0000	*Y* = 1.93487 × 10^6^*X* + 6.14331 × 10^5^	0.072–24	0.9752

**Table 7:** Recovery for the eighteen ginsenosides in ginseng stem-leaf.

Compounds	−ESI PRM		+ESI PRM	
	Recovery (%)	RSD (%) (*n*=6)	Recovery (%)	RSD (%) (*n*=6)
R1	118.32	7.43	103.29	2.03
R2	102.29	5.68	131.23	7.58
Rb1	80.77	6.86	75.38	7.35
Rb2	87.66	3.74	81.24	2.34
Rb3	119.55	5.47	130.08	2.91
Rc	97.41	9.06	84.07	4.05
Rd	111.12	6.75	116.12	3.18
Re	93.25	7.22	78.65	4.32
Rf	76.68	6.51	79.45	5.33
Rg1	95.33	7.75	85.91	4.10
Rg2	90.21	8.09	137.71	4.36
Rg3	101.49	4.73	129.48	10.60
Rh1	112.92	6.18	130.61	4.93
Rh2	103.84	7.90	76.49	2.35
Ro	73.42	6.69	65.42	6.69
F1	80.70	8.16	99.57	4.55
F2	97.93	6.39	78.70	1.80
F11	107.52	4.39	121.05	6.24

**Table 8:** Contents of the eighteen ginsenosides in ginseng stem-leaf.

Content (mg/g)	R1	R2	Rb1	Rb2	Rb3	Rc	Rd	Re	Rg1
FGSL	0.101 ± 0.026	0.078 ± 0.027	0.233 ± 0.063	2.792 ± 0.497	0.289 ± 0.069	1.098 ± 0.184	3.737 ± 0.585	7.394 ± 1.601	2.405 ± 0.593
CGSL	0.214 ± 0.114	0.104 ± 0.075	0.368 ± 0.126	2.635 ± 0.938	0.346 ± 0.192	1.221 ± 0.460	3.390 ± 0.985	8.286 ± 2.364	3.996 ± 1.739
AMGSL	0.391 ± 0.128	0.008 ± 0.002	0.702 ± 0.240	3.543 ± 0.638	4.977 ± 0.481	0.933 ± 0.207	3.424 ± 0.379	8.162 ± 1.635	1.644 ± 0.691

Content (mg/g)	Rg2	Rg3	Rh1	Rh2	Ro	F1	F2	Rf	F11

FGSL	0.396 ± 0.130	0.007 ± 0.002	0.031 ± 0.016	0.003 ± 0.002	0.140 ± 0.070	4.409 ± 0.961	1.067 ± 0.786	0.148 ± 0.050	0.000
CGSL	0.571 ± 0.275	0.006 ± 0.003	0.061 ± 0.049	0.004 ± 0.004	0.087 ± 0.066	4.770 ± 2.490	1.062 ± 0.825	0.160 ± 0.065	0.000
AMGSL	0.899 ± 0.277	0.011 ± 0.003	0.093 ± 0.049	0.005 ± 0.003	0.066 ± 0.088	0.079 ± 0.015	0.921 ± 0.518	0.000	3.271 ± 0.464

**Table 9:** Characterization of analysis markers in CGSL and FGSL based on −ESI full scan MS data.

Variable	Theoretical (*m/z*)	Measured (*m/z*)	Mass error (ppm)	Compounds	Elemental composition	Adduct ions
1117.53@22.49	1117.5284	1117.5286	0.21	—	C_49_H_84_O_25_	[M + HCOO]^−^
1119.58@22.31	1119.5804	1119.5806	0.18	—	C_50_H_90_O_24_	[M + HCOO]^−^
1005.52@12.54	1005.5123	1005.5150	2.78	—	C_44_H_80_O_22_	[M + HCOO]^−^
Rb1	1107.5957	1107.5918	−3.52	Rb1	C_54_H_92_O_23_	[M-H]^−^
Rg1	845.4904	845.4794	−3.54	Rg1	C_42_H_72_O_14_	[M + HCOO]^−^
R1	977.5327	977.5301	−2.66	R1	C_47_H_80_O_18_	[M + HCOO]^−^
975.54@20.84	975.5534	975.5498	−3.69	Vina-ginsenoside R3	C_48_H_82_O_17_	[M + HCOO]^−^
887.49@12.82	887.4857	887.4889	3.78	—	C_40_H_74_O_18_	[M + HCOO]^−^
941.50@25.29	941.4963	941.4987	2.56	—	C_44_H_78_O_21_	[M-H]^−^
1033.55@15.42	1033.5436	1033.5457	2.1	—	C_46_H_84_O_22_	[M + HCOO]^−^
699.42@15.01	699.4266	699.4235	−4.78	—	C_43_H_58_O_5_	[M + HCOO]^−^
1017.51@25.97	1017.5123	1017.5147	2.44	—	C_45_H_80_O_22_	[M + HCOO]^−^
815.47@17.26	815.4798	815.4695	−4.05	Ginsenoside F5	C_41_H_70_O_13_	[M + HCOO]^−^
939.48@25.88	939.4806	939.4844	4.01	—	C_44_H_76_O_21_	[M-H]^−^
1149.56@25.57	1149.5546	1149.5550	0.37	—	C_50_H_88_O_26_	[M + HCOO]^−^
975.51@22.56	975.5018	975.5056	4.12	—	C_43_H_78_O_21_	[M + HCOO]^−^
991.50@15.87	991.4967	991.4990	2.45	—	C_48_H_82_O_18_	[M + HCOO]^−^
957.50@25.45	957.4912	957.4942	3.29	—	C_43_H_76_O_20_	[M + HCOO]^−^
971.51@25.5	971.5068	971.5085	1.78	—	C_44_H_78_O_20_	[M + HCOO]^−^
957.50@24.95	957.4912	957.4941	3.18	—	C_43_H_76_O_20_	[M + HCOO]^−^
961.53@22.27	961.5225	961.5259	3.71	—	C_43_H_80_O_20_	[M + HCOO]^−^
1107.54@20.3	1107.5440	1107.5445	0.43	—	C_49_H_88_O_27_	[M + HCOO]^−^
1093.57@14.55	1093.5648	1093.5688	3.69	—	C_49_H_90_O_26_	[M + HCOO]^−^
973.49@17.4	973.4861	973.4883	2.36	—	C_43_H_76_O_21_	[M + HCOO]^−^
1089.53@24.31	1089.5335	1089.5349	1.32	—	C_49_H_86_O_26_	[M + HCOO]^−^
815.47@18.11	815.4798	815.4777	−2.58	Ginsenoside F3	C_41_H_70_O_13_	[M + HCOO]^−^

## References

[1] Chen Y., Zhao Z., Chen H., Tao Y., Qin M., Liang Z. (2015). Chemical differentiation and quality evaluation of commercial Asian and American ginsengs based on a UHPLC–QTOF/MS/MS metabolomics approach.

[2] Cui S., Wu J., Wang J., Wang X. (2017). Discrimination of American ginseng and Asian ginseng using electronic nose and gas chromatography–mass spectrometry coupled with chemometrics.

[3] Tu T. T., Sharma N., Shin E. J. (2017). Treatment with mountain-cultivated ginseng alleviates trimethyltin-induced cognitive impairments in mice via IL-6-dependent JAK2/STAT3/ERK signaling.

[4] Zhang J., Howard X. Y., Gul W., Pasco D. S., Elsohly M. A., Pugh N. D. (2016). In vitro bioassays for standardization of immune enhancing dietary supplements.

[5] Liu Z., Xia J., Wang C. Z. (2016). Remarkable impact of acidic ginsenosides and organic acids on ginsenoside transformation from fresh ginseng to red ginseng.

[6] Yan B., Wang G., Jiye A. (2007). Construction of the fingerprints of ginseng stem and leaf saponin reference substances and spiked plasma sample by LC-ESI/MS and its application to analyzing the compounds absorbed into blood after oral administration of ginseng stem and leaf saponin in rat.

[7] Wang H., Peng D., Xie J. (2009). Ginseng leaf-stem: bioactive constituents and pharmacological functions.

[8] Cho I. H., Lee H. J., Kim Y. S. (2012). Differences in the volatile compositions of ginseng species (*Panax* sp.).

[9] Bradbury B., Saunders P. (2014). Discrimination of white ginseng origins using multivariate statistical analysis of data sets.

[10] Huang D., Li Y., Zhang M. (2016). Tartaric acid induced conversion of protopanaxadiol to ginsenosides Rg3 and Rg5 and their in situ recoveries by integrated expanded-bed adsorption chromatography.

[11] Huang X., Liu Y., Zhang Y. (2017). Multicomponent assessment and ginsenoside conversions of *Panax quinquefolium* L. roots before and after steaming by HPLC-MS n.

[12] Jia L., Zhao Y., Liang X. J. (2009). Current evaluation of the millennium phytomedicine–ginseng (II): collected chemical entities, modern pharmacology, and clinical applications emanated from traditional Chinese medicine.

[13] Wu W., Song F., Guo D. (2012). Mass spectrometry-based approach in ginseng research: a promising way to metabolomics.

[14] Xia P., Bai Z., Liang T. (2016). High-performance liquid chromatography based chemical fingerprint analysis and chemometric approaches for the identification and distinction of three endangered *Panax* plants in Southeast Asia.

[15] Xie J. (2004). American ginseng leaf: ginsenoside analysis and hypoglycemic activity.

[16] Li Q., Liang X., Zhao L. (2017). UPLC-Q-exactive orbitrap/MS-based lipidomics approach to characterize lipid extracts from bee pollen and their in vitro anti-inflammatory properties.

[17] Renaud J. B., Sabourin L., Topp E., Sumarah M. W. (2017). Spectral counting approach to measure selectivity of high-resolution LC-MS methods for environmental analysis.

[18] Liu F., Ma N., He C. (2017). Qualitative and quantitative analysis of the saponins in *Panax notoginseng* leaves using ultra performance liquid chromatography coupled with time-of-flight tandem mass spectrometry and high performance liquid chromatography coupled with UV detector.

[19] Qi L. W., Wang H. Y., Zhang H., Wang C. Z., Li P., Yuan C. S. (2012). Diagnostic ion filtering to characterize ginseng saponins by rapid liquid chromatography with time-of-flight mass spectrometry.

[20] Wu W., Sun L., Zhang Z., Guo Y., Liu S. (2015). Profiling and multivariate statistical analysis of *Panax ginseng* based on ultra-high-performance liquid chromatography coupled with quadrupole-time-of-flight mass spectrometry.

[21] Goodacre R. (2005). Metabolomics shows the way to new discoveries.

[22] Idle J. R., Gonzalez F. J. (2007). Metabolomics.

[23] Lee D. Y., Kim J. K., Shrestha S. (2013). Quality evaluation of *Panax ginseng* roots using a rapid resolution LC-QTOF/MS-based metabolomics approach.

[24] Guo N., Ablajan K., Fan B., Yan H., Yu Y., Dou D. (2013). Simultaneous determination of seven ginsenosides in Du Shen Tang decoction by rapid resolution liquid chromatography (RRLC) coupled with tandem mass spectrometry.

[25] Mao Q., Yi L. I., Song-Lin L. I., Yang J., Zhang P. H., Wang Q. (2014). Chemical profiles and anticancer effects of saponin fractions of different polarity from the leaves of *Panax notoginseng*.

[26] Li L., Liu S., Ma L., Guo Y., Wang Y., Liu S. (2017). Application of direct analysis in real time-orbitrap mass spectrometry combined with multivariate data analysis for rapid quality assessment of Yuanhu Zhitong Tablet.

[27] Duc N. M., Kasai R., Ohtani K. (1994). Saponins from *Vietnamese ginseng*, *Panax vietnamensis* Ha et Grushv. collected in central Vietnam. II.

[28] Chan T. W. D., But P. P. H., Cheng S. W., Kwok I. M. Y., Lau F. W., Xu H. X. (2000). Differentiation and authentication of *Panax ginseng*, *Panax quinquefolius*, and ginseng products by using HPLC/MS.

